# Dedifferentiated endometrioid adenocarcinoma; clinicopathologic and immunohistochemical features of five cases

**DOI:** 10.4274/jtgga.2017.0090

**Published:** 2018-08-06

**Authors:** Seyran Yiğit, Neşe Ekinci, Leyla Hayrullah, İrfan Öcal, İncim Bezircioğlu

**Affiliations:** 1 Department of Pathology, İzmir Katip Çelebi University, Atatürk Training and Research Hospital, İzmir, Turkey; 2 Department of Obstetrics and Gynecology, İzmir Katip Çelebi University, Atatürk Training and Research Hospital, İzmir, Turkey

**Keywords:** Dedifferentiated endometrioid carcinoma, endometrioid adenocarcinoma, undifferentiated carcinoma

## Abstract

**Objective:**

Dedifferentiated endometrioid adenocarcinoma is a recently defined uterine tumor composed of low-grade endometrioid adenocarcinoma and undifferentiated carcinoma. Herein, we present clinicopathologic, morphologic, and immunohistochemical features of 5 cases of dedifferentiated endometrioid adenocarcinoma.

**Material and Methods:**

All cases which were diagnosed as mixed endometrial adenocarcinoma (endometrioid+undifferentiated carcinoma) or dedifferentiated endometrioid adenocarcinoma between January 2008 and December 2014 were retrieved from the archives of our institution’s pathology department.

**Results:**

The median age of the patients was 58 years. Polypoid growth pattern was seen in 3 patients and 2 were diagnosed at advanced stage. All patients received either external radiotherapy, brachytherapy, chemotherapy or an appropriate combination according to the stage. Only one patient died of the disease. Microscopically, there was a sharp demarcation between the two tumor components. The undifferentiated carcinoma component was composed of diffuse sheets of monomorphic cells lacking any differentiation. Focal pleomorphism and rhabdoid features were also noted. The undifferentiated carcinoma component was variably positive for PAX-8, cytokeratin, EMA, estrogen receptor, and neuroendocrine markers.

**Conclusion:**

Misdiagnosis of undifferentiated carcinoma in dedifferentiated endometrioid adenocarcinoma as grade 3 endometrioid adenocarcinoma is not uncommon. The recognition of morphologic and immunohistochemical features of this newly described entity is crucial because it alters treatment and prognosis.

## Introduction

Uterine and ovarian dedifferentiated endometrioid adenocarcinoma (DEAC) was first described by Silva et al.  in 2006 (1). Based on the definition of the authors, low-grade endometrioid adenocarcinoma (EmC) and undifferentiated carcinoma (UC) are two fundamental elements of this tumor. The low-grade component in these tumors is usually International Federation of Gynecology and Obstetrics (FIGO) grade 1 or 2 EmC. The UC component is characterized by proliferation of medium-sized, homogenous epithelial cells with no glandular differentiation, which grow in a patternless manner and form solid sheets (2). For accurate treatment and prognosis of this neoplasm, a correct pathologic diagnosis is essential (3). Herein, we report five cases of DEAC of the uterus.

## Material and Methods

All cases, which were diagnosed as mixed endometrial adenocarcinoma (EmC+UC) or DEAC between January 2008 and December 2014, were retrieved from the archives of the Pathology Department of our institution. Clinicopathologic data regarding patient age, symptoms, operative procedure, tumor stage (FIGO), lymphovascular invasion, postoperative additional therapies, and survival (months) were assessed. Immunohistochemical (IHC) studies including PAX-8, cytokeratin (CK) AE1-AE3 (epithelial lineage marker, also referred to as ‘keratin’ or ‘pankeratin’), epithelial membrane antigen (EMA) (glandular and ductal epithelial marker, highly expressed by most adenocarcinomas), vimentin (mesenchymal tissue marker), chromogranin A (common neuroendocrine marker), synaptophysin (common neuroendocrine marker), CD56 (common neuroendocrine marker), estrogen receptor (ER) [used to distinguish endocervical (ER-) from endometrial (ER+) adenocarcinoma], and progesterone receptor (PR) (positive in uterine endometrial carcinoma, rules out serous endometrial carcinoma) for routine diagnostic purposes were performed in all cases. Leucocyte common antigen (LCA) (also referred to as CD45, inflammatory and hematopoietic tumor marker), desmin (mesenchymal marker) and CD 99 (small-blue-round-cell tumor marker) were additionally applied to case number 2.

Local ethics committee approval was not sought for this study because it represents a retrospective database review.

## Results

Table 1 illustrates the clinicopathologic features of the cases. The ages of the patients ranged from 54 to 61 years (mean, 58 years). All patients had endometrial biopsies performed because of postmenopausal bleeding. Three patients were diagnosed with grade 1 or 2 endometrioid endometrial adenocarcinoma, others with UC and non-keratinizing squamous cell carcinoma. Total abdominal hysterectomy with bilateral salpingo-oophorectomy (TAH+BSO) and pelvic lymphadenectomy (PL) were performed in all patients.

**Macroscopic findings:** The tumor growth pattern in three cases (cases 1, 3 and 4) was polypoid while the remaining two exhibited infiltrative growth. In cases 2 and 5, cervical involvement and ovarian metastases were also observed. 

**Figure 1:**
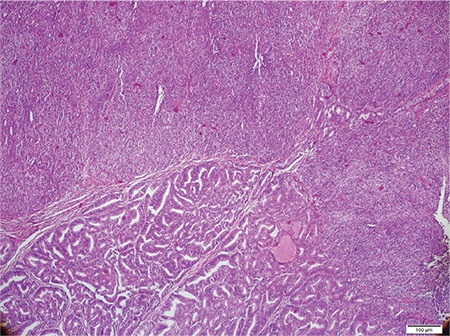
Abrupt transition of low-grade EmC and UC (case #4, Hematoxylin & Eosin, x4) *EmC: Endometrioid adenocarcinoma; UC: Undifferentiated carcinoma*

**Figure 2:**
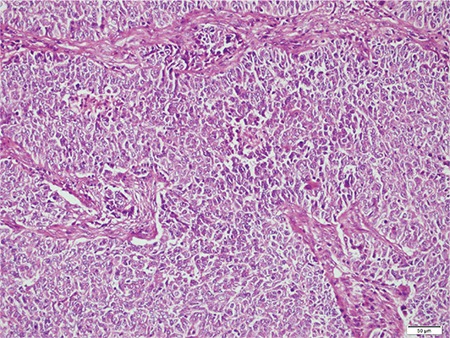
Solid sheets of monotonous cells exhibiting a patternless growth (case #3, Hematoxylin & Eosin, x10)

**Figure 3:**
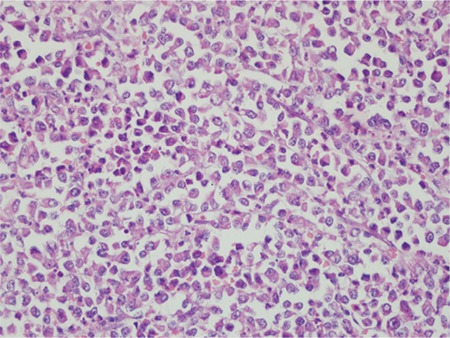
Higher magnification of UC cells showing rhabdoid features (case #2, Hematoxylin & Eosin, x40) *UC: Undifferentiated carcinoma*

**Figure 4:**
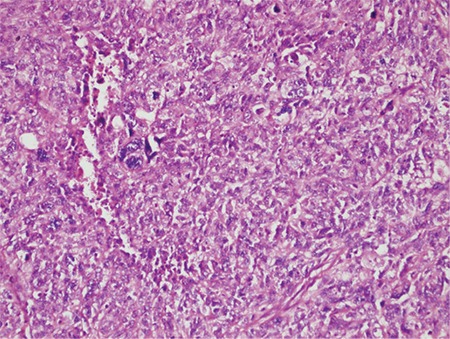
Marked focal pleomorphism in the UC (case #5, Hematoxylin & Eosin, x20) *UC: Undifferentiated carcinoma*

**Microscopic findings:** Tumors in all cases showed sharp demarcation between areas of low-grade EmC and UC (Figure 1, case 4). The undifferentiated component was characterized by solid growth of monomorphic discohesive cells (Figure 2, case 3). Scattered rhabdoid cells and focal marked pleomorphism (cases 2 and 5, respectively) also stood out (Figure 3-4, cases 2 and 5). Four cases exhibited areas of focal or extensive necrosis. All morphologic features are summarized in Table 2. Vascular invasion was only present in case number 2. Lymph node metastases were detected in two patients.

**Figure 5:**
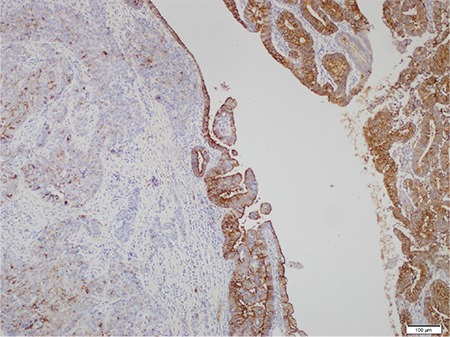
EMA; strong positivity in low-grade EmC component versus patchy, weak staining in the UC component (case #3, x4) *EMA: Epithelial membrane antigen; EmC: Endometrioid adenocarcinoma; UC: Undifferentiated carcinoma*

**Figure 6:**
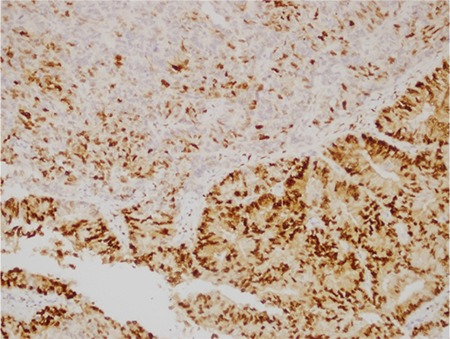
PR; strong nuclear positivity in low-grade EmC component versus sparse nuclear staining in the UC component (case #4, x10) *PR: Progesterone receptor; EmC: Endometrioid adenocarcinoma; UC: Undifferentiated carcinoma*

**Figure 7:**
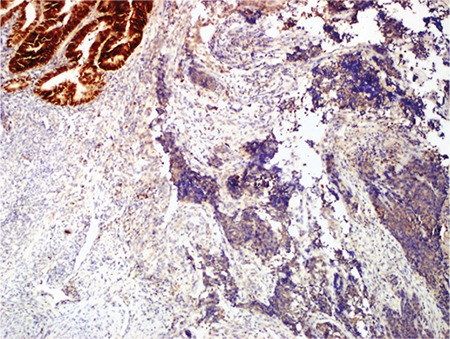
PAX-8 positivity in low-grade EmC and negativity in the UC component (case #1, x20) *EmC: Endometrioid adenocarcinoma; UC: Undifferentiated carcinoma*

IHC features: PAX-8, CK, EMA, ER and PR were strongly and diffusely expressed in the low-grade EmC component (Figure 5, 6, cases 3 and 4), whereas the UC component was diffusely positive for vimentin, focally positive for CK, EMA, and neuroendocrine markers such as synaptophysin, chromogranin A, and CD 56. PAX-8 was negative in UC components of three cases, whereas it was focal positive in two cases (Figure 7, case 1).

Two patients presented with advanced stage disease (FIGO stages III-IV) at the time of diagnosis. Four patients received both radiotherapy (RT) and chemotherapy (CT). All patients but one were still alive as of August 2017.

## Discussion

Uterine EmC is a common neoplasm that is frequently seen in pure form. UC represents 1.6-9% of all endometrial carcinomas (2,3,4). Silva et al. (1) described morphologic features of DEAC in 2006 and it was included in the 2014 version of the book, ‘World Health Organization Classification of the Tumors of Female Reproductive Organs’ (5). Although DEAC is generally presented as case reports, a series of such tumors was recently reported in the literature (6,7,8,9,10,11,12). DEAC primarily occurs during the 6^th^ and 7^th^ decades; consistent with previous studies, the mean age at diagnosis in our study was 58 years (11,12,13,14,15). Similar to existing studies, all patients in our study underwent TAH+BSO and PL (1,6,7,8,9,10,11,12,13,14,15). Advanced FIGO stage of DEAC in the literature is reported to be between 52-92%, whereas in our study, it was found as 40% (1,11,12,13,14). Similar to the cases reported in the literature, all of our patients also received post-operative RT and/or CT (1,8,9,11,12,13,14,15).

Although only a single case was reported to exhibit polypoid growth pattern in studies that described macroscopic features, the main growth pattern in the current study was also found to be polypoid (7,9,10). 

Similar to previous reports, EmC and UC components of the tumors mentioned herein were sharply demarcated from each other and EmC component was either grade 1 or 2 (1,7,9,10,13). The UC component of the current study was characterized by solid sheets of proliferated medium-sized monotonous epithelial cells with no specific pattern, identical to previous reports (2,3,4).

In consonance with the literature, rhabdoid cells, focal pleomorphism, and neuroendocrine differentiation of the DEAC were also noted in some of our cases (1,9,13). Previous studies underscored the use of IHC studies in the diagnosis of DEAC. Even though UC components of DEACs are variably positive for keratins, EMA, and ER, they are mostly negative for PAX-8. In some studies, loss of DNA mismatch repair (MMR) proteins was observed relatively commonly in UC components (1,14). In Stewart and Crook’s study, concordant MMR protein expression in low and undifferentiated components of DEAC was noteworthy (15).

Furthermore, vimentin and focal neuroendocrine marker expressions may be observed in the undifferentiated component. The IHC results of our study are also concordant with the literature except for PAX-8, which was focal positive in 2 of 5 cases (1,9,13). 

Inadvertently, the undifferentiated component in DEAC is often misdiagnosed as grade 3 EmC, serous carcinoma (SC), malignant mixed Mullerian tumor (MMMT), undifferentiated endometrial sarcoma, poorly differentiated neuroendocrine carcinoma or malignant lymphoma (1,9,10,13). 

However, in grade 3 EmC, the tumor cells are morphologically similar to carcinoma cells in glandular areas; solid sheets or nests, and conspicuous glandular structures might also coexist (13). Recent studies showed inactivation of SWI/SNF complex subunits such as INI1 (SMARCB1), BRG1 (SMARCA4) and ARID1A (BAF250a) whose alterations might help distinguish poorly (grade 3) differentiated endometrial carcinoma from DEAC (15,16). 

In SC glandular component with papillary features, slit-like lumens, background endometrial atrophy and architectural-cytological discordance can also support the diagnosis. MMMT is a biphasic tumor composed of high-grade carcinoma, usually serous carcinoma, and a sarcomatous component that is typically reminiscent of a pleomorphic sarcoma (9,13). Undifferentiated endometrial sarcomas are composed of more pleomorphic cells and focally spindled cells (14). Neuroendocrine carcinoma and malignant lymphoma can be differentiated on the basis of their specific IHC features in the absence of well-differentiated endometrioid carcinoma (13,14). Extensive sampling, high awareness of the morphologic characteristics of this tumor and IHC studies are essential for accurate diagnosis. 

Follow up studies revealed that DEAC is a much more aggressive tumor than grade 3 EmC (1,13). Due to the small number of patients and the short follow-up period, the non-aggressive tumor behavior present in our study prevents us from reaching a similar conclusion. POLE mutations are associated with a favorable prognosis; however, we were unable to perform molecular analysis in our study (17). 

In conclusion, DEAC is a rare, but most frequently misdiagnosed aggressive tumor. Due to variable therapeutic approaches and prognostic implications, identifying and correctly diagnosing DEAC in the endometrium is crucial.

## Figures and Tables

**Table 1:**
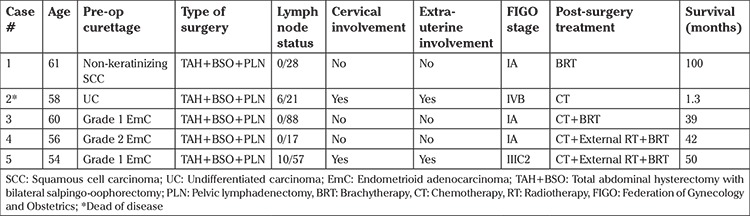
Clinicopathologic features of the cases

**Table 2:**
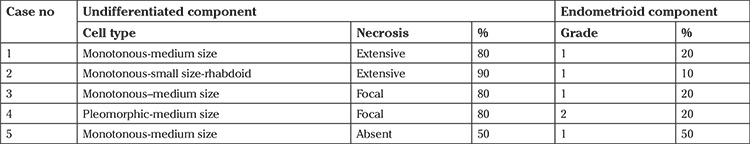
Morphologic features of the tumor components
